# Total or partial tonsillar resection (tonsillectomy or tonsillotomy) to change the quality of life for adults with recurrent or chronic tonsillitis: study protocol for a randomised controlled trial

**DOI:** 10.1186/s13063-021-05539-4

**Published:** 2021-09-15

**Authors:** Aleksi Laajala, Paulus Tokola, Timo J. Autio, Timo Koskenkorva, Mikko Tastula, Pasi Ohtonen, Esa Läärä, Olli-Pekka Alho

**Affiliations:** 1 Department of Otorhinolaryngology and Head and Neck Surgery, Oulu University Hospital, P.O. Box 5000, FIN-90014 Oulu, Finland; 2 PEDEGO Research Unit, University of Oulu, Oulu, Finland; 3 Medical Research Center Oulu, Oulu, Finland; 4 Division of Operative Care, Oulu University Hospital, Oulu, Finland; 5 Research Unit of Mathematical Sciences, University of Oulu, Oulu, Finland

**Keywords:** Chronic tonsillitis, Recurrent tonsillitis, Quality of life, Treatment, Tonsillectomy, Tonsillotomy, Randomised controlled trial

## Abstract

**Background:**

Tonsillar surgery has been used for decades to treat recurrent and chronic tonsillitis in adults. Recurrent and chronic tonsillitis result in disturbing symptoms, treatment costs, sick leave, and impaired quality of life (QoL). Theoretically, removing all or part of the altered pathological palatal lymphoid tissue alleviates the symptoms and enhances the QoL. Whether this is true with total or partial tonsillar resection (tonsillectomy (TE) and tonsillotomy (TT), respectively) has not been reported in a randomised trial yet.

**Methods:**

We conduct a multicentre, partly blinded, randomised, 6-month, parallel-group clinical study including 285 adult participants referred to surgical treatment for chronic or recurrent tonsillitis. The participants will either have TE, TT or watchful waiting (WW). The primary outcome will be the difference between the mean disease-specific Tonsillectomy Outcome Inventory-14 (QoL questionnaire) scores at 6 months. Comparison is made firstly between the combined TE+TT and WW groups (superiority analysis), and secondly between the TE and TT groups (non-inferiority analysis).

**Discussion:**

This study will add significant new information to the effects and harms of TE and TT procedures in the treatment of adults with chronic or recurrent tonsillitis.

**Trial registration:**

ClinicalTrials.gov: NCT04657549

**Supplementary Information:**

The online version contains supplementary material available at 10.1186/s13063-021-05539-4.

## Administrative information

The order of the items has been modified to group similar items (see http://www.equator-network.org/reporting-guidelines/spirit-2013-statement-defining-standard-protocol-items-for-clinical-trials/).

**Table:** 

Title {1}	Total or partial tonsillar resection (tonsillectomy or tonsillotomy) to improve quality of life for adults with recurrent or chronic tonsillitis
Trial registration {2a and 2b}.	ClinicalTrials.gov registration number NCT04657549
Protocol version {3}	14.4.2021 version number 3
Funding {4}	Governmental research grant No external funding
Author details {5a}	Laajala A, MD, doctoral student, specialist in Otorhinolaryngology Tokola P, MD, doctoral student, specialist in Otorhinolaryngology Autio T, MD, PhD, specialist in OtorhinolaryngologyKoskenkorva T, MD, PhD, specialist in Otorhinolaryngology Tastula M, MD, specialist in Otorhinolaryngology Ohtonen P, MSc, statistician Läärä E, professor of Statistics Alho O-P, MD, PhD, professor of Otorhinolaryngology Department of Otorhinolaryngology and Head and Neck Surgery, Oulu University Hospital, Oulu, Finland PEDEGO Research Unit, University of Oulu, Finland Medical Research Center Oulu, Oulu, Finland Division of Operative Care, Oulu University Hospital, Oulu, Finland Research Unit of Mathematical Sciences, University of Oulu, Finland
Name and contact information for the trial sponsor {5b}	Trial Sponsor: Oulu University Hospital Sponsor's Reference: Y-0679480-9 Contact name: Ms. Minna Mäkiniemi Address: P O Box 10, FIN-90029 Oulu University Hospital, Finland Telephone: +358 8 315 2011 Email: minna.makiniemi@ppshp.fi
Role of sponsor {5c}	The funders have no role in the design, data collection and analysis, the decision to publish or the preparation of the manuscript

## Introduction

### Background and rationale {6a}

Chronic and recurrent tonsillitis are relatively common in adult populations worldwide, their exact occurrence depending on the definition. Recurrent tonsillitis is typically defined as a minimum number of tonsillitis bouts in a given time period. Episodes should involve the palatine tonsils based on signs found during the episodes (e.g. tonsillar oedema or erythema, exudative tonsillitis, anterior cervical lymphadenitis). Chronic tonsillitis is mainly defined as having throat pain for a prolonged time period. In addition, at least one symptom or sign should indicate that symptoms originate from the palatal tonsils (disturbing tonsil stones, halitosis, anterior cervical lymphadenitis, tonsillar exudates, abnormal tonsillar crypts).

Patients with chronic and recurrent tonsillitis face disturbing symptoms, significant financial burden in the form of recurrent absences from work, health care visits, medical treatment costs and social harms [1], thus impairing their quality of life (QoL). Tonsillar diseases have been observed to lower both disease-specific and generic QoL [2, 3].

The conservative treatment for recurrent tonsillitis episodes mainly involves antibiotic courses and analgesics. Chronic tonsillitis may be treated conservatively with analgesics, mouth rinses and mechanical removal of tonsil stones by the patients themselves (e.g. with cotton swabs). In adults, several guidelines suggest that tonsil surgery may be used, particularly for recurrent tonsillitis [4]. Still, the current practice of surgical treatment of these conditions in adults is largely unclear, and there have been considerable variations in the surgical rate between different countries [5, 6]. The question remains of whether to have tonsillar surgery at all and, if so, whether to do a lighter tonsillotomy (TT) or larger tonsillectomy (TE). This is due to the lack of scientific evidence in tonsillar surgery on chronic and recurrent tonsillitis.

TE refers to subcapsular dissection of the tonsillar tissue and its encasing fibrous capsule. TT refers to removal of a variable volume of tonsillar lymphoid tissue leaving the lateral fibrous capsule intact. Theoretically, removal of all or part of the altered pathological palatal lymphoid tissue alleviates the patients’ symptoms. This should reduce the chronic throat symptoms and the number and severity of recurrent episodes improving the QoL of the patients. The possible harms related to TT and TE include postoperative pain, haemorrhage, infections, rare anaesthetic complications and even death. In TT, leaving the capsule intact protects the underlying muscles and larger diameter blood vessels, theoretically minimising perioperative complications.

Our research group has shown in two randomised controlled trials that, in adults with recurrent tonsillitis, the risk of further episodes diminished after TE as compared to watchful waiting. Two randomised studies have reported that TT and TE both enhanced the generic QoL [7] and disease-specific QoL [8]8 in adults with infective or obstructive tonsillar disease. These latter studies lacked a control group and were relatively small and underpowered for non-inferiority analyses. Several randomised trials involving obstructive and infective tonsillar diseases in adults demonstrated that TT involves significantly fewer postoperative harms than TE [7–9].

Overall scientific evidence for tonsil surgery improving QoL among adults suffering from chronic or recurrent tonsillitis is insufficient. Thus, a randomised controlled trial is needed to compare the QoL benefits and harms related to TE and TT and watchful waiting among adult patients with recurrent or chronic tonsillitis.

### Objectives {7}

Our main objective is to determine whether tonsil surgery improves the QoL in adult patients with recurrent or chronic tonsillitis compared to WW and whether lighter TT is as effective as TE. Our hypothesis is that, among these patients, both TE and TT are more effective than WW in enhancing QoL without significant risks (superiority assumption) and that TT is non-inferior to TE when the surgical groups are compared (non-inferiority assumption).

### Trial design {8}

**Fig. 1:**
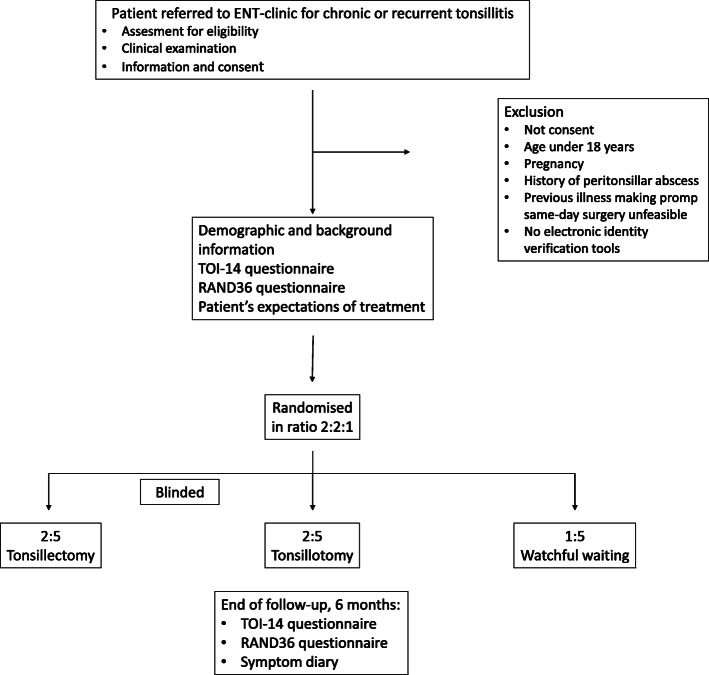
The study flowchart

This study is designed as a multicentre, randomised, controlled, parallel-group, partly blinded trial with a follow-up period of 6 months. Participants will be assigned to three groups using block randomisation: tonsillectomy group (TE), tonsillotomy group (TT) and control group with watchful waiting (WW) in ratio 2:2:1. Fig. 1.

## Methods: Participants, interventions and outcomes

### Study setting {9}

The study is being conducted at one university hospital (Oulu University Hospital, tertiary care) and four central hospitals (Lapland Central Hospital, Länsi-Pohja Central Hospital, Keski-Pohjanmaa Central Hospital and Seinäjoki Central Hospital, all secondary care) in Finland. We will recruit patients referred to these hospitals’ outpatient ear, nose and throat clinics. All cases come from a combined population of 857,784 inhabitants in Finland. This is the secondary care area of these five hospitals and comprises 78 municipalities that maintain one primary health centre each. Almost all tonsillar surgeries in the area are done in these hospitals. The health care system in Finland is based on a general health insurance scheme and provides equal access to medical services for all citizens. All patients must first present in primary care before referral to secondary or tertiary care.

### Eligibility criteria {10}

#### Inclusion criteria

The inclusion criteria regarding recurrent tonsillitis are as follows:

- At least three tonsillitis episodes in 6 months or four episodes in 12 months.
- These episodes must be disabling, prevent normal functioning and be severe enough for the patient to seek medical attention.
- Episodes must be thought to involve the palatine tonsils based on signs found during the episodes (e.g. tonsillar oedema or erythema, exudative tonsillitis, anterior cervical lymphadenitis).
- No throat cultures or antigen tests to show infection with group A *Streptococcus* are needed.

The inclusion criteria regarding chronic tonsillitis are as follows:

- Recurrent or chronic throat pain for at least 6 months.
- At least one symptom or sign must indicate that symptoms originate from the palatal tonsils (disturbing tonsil stones, halitosis, anterior cervical lymphadenitis, tonsillar exudates, abnormal tonsillar crypts).
- Symptomatic treatment has not been effective.

#### Exclusion criteria

Potential participants have to be excluded in the following cases:

- Age under 18 years
- Pregnancy
- History of peritonsillar abscess
- Previous illness making prompt same-day surgery unfeasible
- No electronic identity verification tools.

The present criteria for recurrent and chronic tonsillitis are modified criteria presented in the Northern Tonsilla Registry [6].

### Who will obtain informed consent? {26a}

The participants will be recruited from consecutive adult patients referred to the ear, nose and throat outpatient department at the five participating hospitals because of chronic throat problems. Altogether, 14 research members from the ear, nose and throat departments at the participating hospitals do the recruitment. Patients will be screened for study participation based on the inclusion and exclusion criteria. The participants are interviewed, and written evidence of previous tonsillitis episodes is looked for from referral letters and patient files. Other clinical criteria are requested from the participants. Those who fulfil the eligibility criteria and express an interest in participating in the study will be given a verbal explanation of the study details and the written consent form, and any questions regarding the study will be answered. Then, each participant will have sufficient time to decide whether to participate in this study. For those willing to participate, written consent will be obtained. For those who fulfil the criteria but decline to participate, age, gender, diagnosis (recurrent or chronic tonsillitis) and reason for refusal, if given, are recorded.

### Additional consent provisions for collection and use of participant data and biological specimens {26b}

Participants will be asked if they agree to the use of their data on the consent form, should they choose to withdraw from the trial. This trial does not involve collecting biological specimens for storage.

### Interventions

#### Explanation for the choice of comparators {6b}

We compare the conservative treatment of recurrent and chronic tonsillitis to TE or TT. Most of the current national guidelines for adults recommend conservative treatment as the primary mode of treatment if the number of bouts of tonsillitis is less than 5 to 7 episodes per year for the first year [4, 10]. Using usual care as a comparator ensures that the effects of TE and TT are not over- or underestimated and that the patients are more willing to participate.

#### Intervention description {11a}

Before randomisation, all participants are given standardised information on the trial and surgical procedures and postoperative care by means of a video and written text. The patients randomised to the surgical groups are blinded to the operation type, so the information only mentions tonsillar surgery. The material includes information on the anaesthesia, length of hospital stay, risks related to the operation (throat pain, bleeding, fever, globus feeling) and length of sick leave. Moreover, instructions on postoperative nutrition, physical activity, wash-up and teeth-brushing are given. Information on when to contact the treating hospital and contact details are also provided.

TE and TT will be performed as day surgery under general anaesthesia. The residents and specialists of ear, nose and throat diseases at the participating hospitals will perform the operations. Patients in the TE group (extracapsular dissection tonsillectomy) will undergo a subcapsular dissection of the tonsillar tissue and its encasing fibrous capsule away from the lateral pharyngeal muscles. This is done using cold-steel dissection or with electrocautery (bipolar or monopolar) or coblation technology. Patients in the TT group (intracapsular dissection tonsillectomy) will have most of the tonsillar lymphoid tissue removed, leaving the lateral fibrous capsule intact. TT is performed with dissection, microdebrider, electrocautery or coblation technology. In TT, the underlying superior pharyngeal constrictor muscles and larger diameter blood vessels are protected, which theoretically minimises perioperative complications.

Postoperatively, according to the advice given in the video and written material, the patients must avoid hard and hot food for 2–4 days, sauna and hard physical activity for 2 weeks and ingest liquids often to prevent the throat from drying. At first, patients are told to take pain medication regularly and later on if needed. Pain medication includes oxycodone/naloxone hydrochloride 5/2.5 mg 1–2 tablets twice a day, paracetamol 1 g one tablet three times a day and dexketoprofen 25 mg tablet three times a day. Sick leave from 1 to 2 weeks is given depending on the patient’s occupation. Finally, the patients are given the hospital’s contact details and are advised to make contact in case of high fever, deteriorating general condition or significant bleeding from the throat.

Patients in the WW group will continue the conservative treatment with antibiotics and analgesics for acute symptoms when appropriate and analgesics, mouth rinses and mechanical removal of tonsil stones for chronic symptoms as they choose. The details of the individual participant’s treatment will be decided according to the attending clinician’s judgement and Finnish clinical guidelines [11].

#### Criteria for discontinuing or modifying allocated interventions {11b}

The assigned study intervention may be discontinued, mainly for withdrawal of participant consent. The participant may refuse to have the assigned surgery or discontinue the conservative treatment, which serves as a control. The group assigned to TT or TE will be operated on within 3 weeks of enrolment, so by paying attention to the proper criteria for study entry, we will minimise the risk of refusal. Similarly, the group assigned to conservative treatment will eventually be operated on after 6 months of follow-up when this trial is over. The waiting time here, which is within the normal limits for our hospital, decreases the risk of participants seeking the operation elsewhere. In addition, all participants receive a reminder call 3 months after enrolment to ensure they continue their participation in the study. For these reasons, no standard criteria for discontinuations are designed. If participants discontinue their assigned intervention, we still aim to collect the outcome data as planned to prevent missing data.

#### Strategies to improve adherence to interventions {11c}

We use the following ways to limit missing data in the design and conduct of this trial [12]. Prior to the study, the target population of our referred patients has not been adequately served by treatments, so has an incentive to remain in the study. We allow the control group a flexible treatment regimen that accommodates individual differences in efficacy and side effects to reduce the dropout rate because of lack of efficacy or tolerability. The follow-up period for the primary outcome will be relatively short. We will select investigators who have a good track record in enrolling and following participants and collecting complete data in previous trials. All investigators in other hospitals will be contacted regularly and informed about study progress. We will set 10% as an acceptable target rate for missing data concerning the primary outcome and will monitor the trial’s progress with respect to this target. We will limit the burden and inconvenience of data collection on the participants using a mobile phone application and make the study experience as positive as possible. We will emphasise to the investigators and study staff that keeping participants in the trial until the end is essential, regardless of whether they continue to receive the assigned treatment. We will also give this information to study participants. We will keep contact information for participants up to date. All participants will receive a call at three months follow-up to remind them of data collection as well as their treatment plan.

#### Relevant concomitant care permitted or prohibited during the trial {11d}

During the follow-up period, the participants in all three groups are allowed standard treatment of tonsillitis episodes, including antibiotics and analgesics. These medications are chosen by the patients’ primary physicians as needed. Similarly, chronic throat pain may be treated with analgesics, mouth rinses or mechanical cleaning of the tonsils as the patients choose.

#### Provisions for post-trial care {30}

There is no anticipated harm or compensation related specifically to trial participation. The assigned treatments are ordinary, and the regular malpractice insurance covers the participants.

#### Outcomes {12}

We aim to collect all data electronically with the paper and pencil method being used only as a backup method if electronic data collection fails. The enrolling physician will add each patient to a CureLisa randomisation database and Terveyskylä database [13, 14]. The CureLisa randomisation programme and database are commercial tools for scientific use. Terveyskylä is a Finnish public web service for special health care. It provides information, support and online treatment for patients and various tools for professionals, including data collection web pages and mobile application for scientific purposes.

The enrolling physician adds the following information to the CureLisa database: demographic data, main indication for surgery (recurrent or chronic tonsillitis) and details on clinical parameters. Furthermore, physical findings at enrolment and surgical details are recorded. CureLisa generates an identity code for the patient and randomises him/her according to the allocation lists. The patient logs in to the Terveyskylä service by electronic identity verification tools and fills in contact details, demographic and background information on prior illnesses and past and current symptoms. In addition, data on the patient’s expectations for the treatment and initial reasons for seeking medical care are collected.

The following outcome data are gathered using CureLisa and Terveyskylä services: surgical complications, medical visits and antibiotic courses for throat-related reasons. Participants keep a symptom diary with their mobile phones using the Terveyskylä mobile application. They grade (from 0=no to 10 very severe) daily their acute throat-related symptoms, use of analgesics and absence from work or study.

To record the quality of life, we use the disease-specific Tonsillectomy Outcome Inventory – 14 (TOI-14) and general Research and Development 36-item Health Survey (RAND-36) questionnaires, both filled in by the patient using the Terveyskylä service. The TOI-14 questionnaire was initially developed and validated in the German language for adults with chronic tonsillitis [1]. This disease-specific QoL instrument comprises 14 questions, which assess the effect of various aspects of throat-related illnesses on patients’ lives. The questions are divided into four subscales: throat-related problems, overall health, resources and psychosocial restrictions. The questions particularly concern the past 6 months of the patients’ lives. The patient answers each question using Likert scales (0= no problem to 5= most severe problem). The sum score is formed by adding up the answers, dividing this sum by 70 and multiplying this by 100 to give an adjusted score out of 100 (maximum). The higher the score, the poorer the throat-related QoL. We have previously translated, culturally adapted and validated the Finnish TOI-14 instrument according to the recommendations of the International Society for Quality of Life Research (ISOQOL) and Consensus-based Standards for the Selection of Health Measurement Instruments (COSMIN) initiative [15, 16]. According to standards for a QoL questionnaire set out by ISOQOL, the Finnish TOI-14 had good psychometric properties. The conceptual and measurement model was meaningful and the instrument showed good reliability, content and construct validity, as well as responsiveness [17].

RAND-36 is a short-form health survey developed as a tool for outcome measurement in the Medical Outcomes Study [18]. RAND-36 is divided into eight domains, which measure generic health-related QoL. The domains are physical functioning, role limitations due to physical health or emotional problems, energy/fatigue, emotional well-being, social functioning, pain and general health. The instrument’s scoring algorithm produces eight individual values between 0 and 100 for each domain, with higher scores indicating better QoL. We use the Finnish translations of this instrument, which has been translated, culturally adapted and validated [19].

The complete list of the collected variables (variable name, type, collection time and method and variable scale and values) is given in Additional file 1.

### Primary outcome

TOI-14 follow-up score at the end of 5 to 6 months of follow-up.

The primary analysis has two phases. Firstly, the TOI-14 score in the combined surgical group (TT+TE) is compared to that in the WW group. Secondly, the score in the TT group is compared to that in the TE group.

### Secondary outcomes

1. Difference in RAND-36 domains scores at the end of follow-up between groups.
2. Difference in proportions of participants benefiting clinically significantly from the intervention between the groups (minimum important change in TOI-14 score) at the end of follow-up.
3. Difference in the numbers of days patients have throat pain, bad breath, bleeding from the throat, bothering tonsil stones (in all severity scaled 0–10) between the groups during the follow-up.
4. Difference in the number of days patients take dexetoprofen 25 mg, acetaminophen 1 g, or oxycodone/naloxone 5mg/2.5mg and amount of pain medication due to throat pain between the groups during the follow-up.
5. Difference in the numbers of medical visits, antibiotic courses and days with absence from work or study for throat symptoms between the groups during the follow-up.
6. Difference in proportions having feeling of tightness/globus in throat, voice problems and mandibular joint problems between the groups during the follow-up.
7. Frequency of postoperative pain, bleeding, infections, dental injury and anaesthetic complications in the surgical groups during the follow-up.

As recurrent and chronic tonsillitis mainly lowers QoL, an effective treatment should primarily enhance it without substantial harms. Therefore, we chose the disease-specific quality of life change after TE and TT as our primary outcome and recorded the clinically relevant possible harms as secondary outcomes.

#### Participant timeline {13}

Participants in the surgical groups (TE and TE) will be operated on as soon as practically possible, which we estimate to be within 3 weeks of assignment. The participants in the control group will be placed on a waiting list to undergo tonsillar surgery after 5 to 6 months after the end of this trial. Five to 6 months is the usual operational delay for elective surgery in our clinics so, for the control group, follow-up will finish before the participants are operated on.

**Fig. 2:**
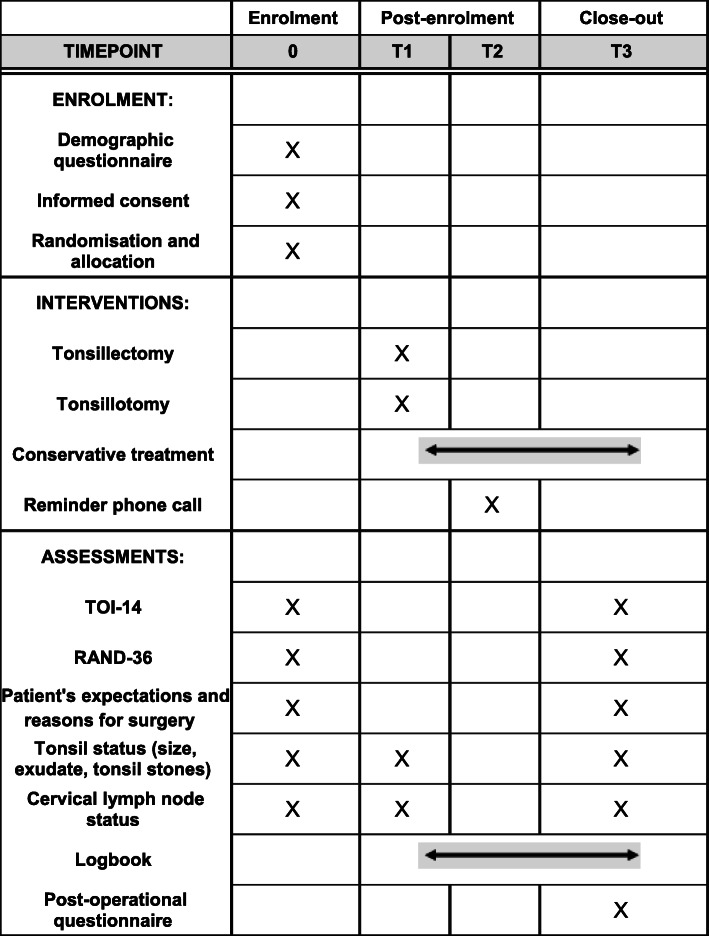
The schedule of enrolment, interventions and assessments. T1 = within 2 to 3 weeks of enrolment, T2 = 3 months follow-up, and T3 = 5–6 months. The study logbook is filled continuously in a mobile phone application between surgery and the end of follow-up for surgical groups and between enrolment and the end of follow-up for the control group. TOI-14 = Tonsillectomy Outcome Inventory – 14, RAND-36 = Research and Development 36-item Health Survey.

The enrolment, interventions, assessments and study visits of our trial are presented in Fig. 2.

#### Sample size {14}

Our principal outcome is a disease-specific QoL questionnaire TOI-14 score at 6 months of follow-up. According to Laajala et al. [17], a difference of 10 points is clinically significant. Further, the TOI-14 score was detected to be highly skewed to the right with excess zeroes at 6 months of follow-up, so we used a log-transformation (log (1+TOI-14)) in sample size calculations. Our hypotheses were (1) both surgically treated groups (TE+TT) were superior (1.50 vs 2.71, SD=1.0) compared to the follow-up (WW), and (2) TT is non-inferior to TE (1.50 vs 1.50, SD=1.0 with non-inferiority marginal=0.41). In both calculation *α*=0.05 and *β*=0.10 (power=0.90). According to this, (1) 15 patients and (2) 102 patients per group will be needed. To ensure that we have adequate power for the follow-up group, we decided to recruit 51 patients into that group. Further assuming a drop-out rate of 10%, the sample size for surgically treated groups is 114 and for the follow-up group 57 patients (altogether 285). Sample size estimation was performed only for the principal outcome, and other comparisons are hypothesis generating only.

#### Recruitment {15}

Altogether, 14 research members from the departments of ear, nose and throat in the participating five hospitals will be in charge of the recruitment process. We expect the contested sample size of 285 participants to be recruited by 2023. The research team will follow-up the actualised recruitment rate regularly during the bimonthly meetings. No financial or non-financial incentives are provided to trial investigators or participants for enrolment.

### Assignment of interventions: allocation

#### Sequence generation {16a}

Computer-based random allocation lists are created, one for Oulu University Hospital and another for the remaining four centres and a separate list for recurrent and chronic tonsillitis. Patients will be allocated to TE, TT and WW in ratio 2:2:1. Random permuted blocks will be used (block size varying between 5 and 10). Only statistician who created the list is aware of the allocation order.

#### Concealment mechanism {16b}

The allocation sequence will be concealed from the investigators enrolling participants using the centralised online randomisation service, CureLisa.

#### Implementation {16c}

A biostatistician not involved in the assignment or care of the trial participants generates the randomisation sequence with a computerised random number generator. Participants who fulfil the inclusion criteria will be recruited by the ear, nose and throat residents and specialists involved in the trial, who will only receive the randomised allocation group for each participant after recruitment and will not have access to the allocation list.

### Assignment of interventions: blinding

#### Who will be blinded {17a}

Trial participants who are randomised to either the TE or TT group are blinded to which of these two surgical treatments they are receiving. The preoperative information about the surgery does not reveal which operation the participants randomised in the two surgical groups are receiving. For a non-professional, it is practically impossible to conclude, by looking at the throat specifically postoperatively, whether a total or partial resection of palatal tonsils has been performed. We conceal this information from the medical charts concerning the study treatment on each participant hospitals’ databases as well as from the National Health Archive database (Kanta). To test the success of the blinding procedure, we asked the participants in the TE and TT groups at the end of follow-up which of the procedures they think they had had and why they thought so. We regard the blinding to have failed, if the participants’ sensitivity and specificity values pointing to the correct procedure both exceed a value of 0.75.

#### Procedure for unblinding if needed {17b}

The need for unblinding is highly unlikely, because postoperative treatment and complications are similar in TT and TE. Nevertheless, if there is a need for unblinding, investigators can unblind a patient’s group from their own hospital. Each hospital keeps a sealed record of their patients’ ID number and allocated groups.

### Methods: Data collection, management and analysis

#### Plans for assessment and collection of outcomes, baseline and other data {18a}

Study candidates are evaluated at the ear, nose and throat outpatient departments of the participating five hospitals by the trial investigators. Data from interviews, referral letters and patient files are used to evaluate whether the trial candidates fulfil the eligibility criteria. Enrolling physicians collect part of the baseline information using the randomisation service, CureLisa. The rest is gathered with an online questionnaire using the Terveyskylä service, which the participant patients fill in. Outcome data is collected similarly online by the patients in the Terveyskylä service. It includes data on medical visits, sick leave and various symptoms during the follow-up. The patients fill in the TOI-14 and RAND-36 questionnaires at baseline and at the end of follow-up. The Finnish versions of the quality-of-life instruments have been found to be reliable, valid and responsive [17, 19]. Possible surgical complications are collected at the potential postoperative visits and contacts and at the end of the follow-up from the online databases and patient files. For the complete list of variables collected, see Additional file 1.

#### Plans to promote participant retention and complete follow-up {18b}

We stress the importance of this trial to participating patients and the physicians treating them, and call participants to remind them to fill the questionnaires and symptom diary in the databases and about the follow-up visit. At the follow-up visit, we will provide blinded group information about which operation they had. We will ensure that all randomised participants fill the QoL questionnaires at baseline, and at the end of follow-up, if they have not done so in the Terveyskylä service. For those participants who do not show up to the follow-up visit, we shall try to get answers to these questionnaires by phone to avoid missing outcome data. All cases are analysed on an intention-to-treat principle. Participants may withdraw from the study at any point. The reason for withdrawal is recorded if the participant so allows.

#### Data management {19}

We use electronic data capture. The data are coded at entry. To reduce errors, we use online data entry forms that are as clear as possible, questions with answer options visible whenever possible and a rule that prevents one from proceeding until all questions have been answered. Data integrity will be enforced with referential data rules, valid values, range checks and consistency checks against data already stored in the database. From the CureLisa and Terveyskylä databases, the data will be transferred automatically to the SPSS file where it is analysed. Two authors (A.L., P.T.) will create a coded IBM SPSS file for all collected data, which is then commented on and revised by the whole research group. The final SPSS data file is checked using the following methods: verification that the data are in the proper format or within an expected range of values, and independent source document verification of a random subset of data.

#### Confidentiality {27}

Participants’ confidentiality is secured by (1) the creation of coded, depersonalised data where the participant’s identifying information is replaced by an unrelated sequence of characters; (2) secure maintenance of the data and the linking code in separate locations using encrypted digital files within password protected folders and storage media; and (3) limiting access to the minimum number of individuals necessary for quality control, audit and analysis (A.L., P.T., P.O., E.L., O.-P.A.). Participant files will be stored for 3 years after the completion of the study. The data are not transmitted elsewhere.

#### Plans for collection, laboratory evaluation and storage of biological specimens for genetic or molecular analysis in this trial/future use {33}

This trial does not involve the collecting, laboratory evaluation or storage of biological specimens for genetic or molecular analysis.

### Statistical methods

#### Statistical methods for primary and secondary outcomes {20a}

To describe the data the following methods will be used. For variables, whose distribution can reasonably be approximated by the Gaussian distribution, the results will be summarised by the mean and standard deviation (SD). Variables for skewed distributions will be described as median and interquartile range. Categorical variables will be expressed as frequencies with percentages.

The primary and secondary outcomes, measures and planned statistical analyses are displayed in Table 1. Based on our earlier study [17], the principal outcome, TOI-14 score at 6 months of follow-up, will most probably be left-truncated at zero and right-skewed, so tobit-analysis is used with log (1+y) transformation [20, 21]. The primary analysis has two phases. Firstly, the TOI-14 score at 6 months of follow-up in the combined surgical group (TT+TE) is compared to that in the WW group. Secondly, the score in the TT group is compared to that in the TE group. Effects will be estimated by adjusted mean differences in the log-transformed scores with 95% confidence intervals. Based on our earlier observational studies on the subject [17, 22], the following covariates are included in the multivariable adjusted tobit-model: gender and baseline TOI-14 score together with stratification factors: the enrolling centre (Oulu vs. others) and main complaint (recurrent vs. chronic tonsillitis). The analyses will be performed on an intention-to-treat basis. Per protocol analysis will be performed as sensitivity analysis, and the results from comparisons on secondary outcomes will be used to generate hypothesis for future trials.

**Table 1:** Variables, measures and planned statistical analyses

Variable/Outcome	Hypothesis	Outcome measure	Method of analysis
Primary outcome	TE and TT improve TOI-14 scores at 6 months as compared to WW	Difference between the mean TOI-14 scores of combined TE+TT and WW group	Estimation of differences between log transformed means based on tobit model and adjusting for selected covariates (adjusted sex, baseline TOI-14 score, enrolling center (Oulu vs. others) and main complaint (recurrent vs. chronic tonsillitis)
	TT is non-inferior to TE in improving the TOI-14 scores	Difference between the mean TOI-14 scores of TT and TE group	
Secondary outcomes			
General QoL change	Improvement occurs	Difference between the mean RAND 36 scores at 6 months in TE+TT vs. WW groups and in TE *vs.* TT group	Analysis of covariance
Proportion benefiting	Improvement occurs	Difference in proportions benefiting (TOI-14 > MIC) in TE+TT group *vs.* WW group and in TE *vs.* TT group	Estimation of risk ratio, risk difference and number needed to treat (with 95 % CI), NNT
No. of episodes, visits, antibiotic courses, sick days	Improvement occurs	Difference in medical visits, antibiotic courses and sick days in TE+TT group *vs.* WW group and in TE *vs.* TT group	Difference in means with 95% CI
No. of symptomatic days	Improvement occurs	Difference in no. of days with various harmful symptoms and with various analgesics taken in TE+TT group *vs.* WW group and in TE *vs.* TT group	Difference in means with 95% CI
No. of harmful symptoms	Improvement occurs	Difference in proportions of having various harmful symptoms in TE+TT group *vs.* WW group and in TE *vs.* TT group	Chi-squared test, Risk ratio with 95% CI
No. of postoperative complications	Improvement occurs	Frequency of postoperative pain, bleeding, infections, dental injury and anaesthetic complications	Number (%)

#### Interim analyses {21b}

No interim analyses will be done.

#### Methods for additional analyses (e.g. subgroup and adjusted analyses) {20b}

We plan to conduct one subgroup analysis. From our prior research, we know that patients with recurrent tonsillitis have worse QoL scores than those with chronic tonsillitis, so we compare the results from the primary analysis in these two conditions. We anticipate that the effect of surgery (TE+TT) on TOI-14 scores as compared to WW will be larger in recurrent tonsillitis than in chronic tonsillitis. Results from this subgroup analysis will be used to generate hypothesis for future trials.

As described above, the analysis of the primary outcome will be adjusted where gender, baseline TOI-14 score together with stratification factors will be used as covariates.

#### Methods in analysis to handle protocol non-adherence and any statistical methods to handle missing data {20c}

An “as randomised” analysis is performed, which retains participants in the group to which they were originally allocated (intention-to-treat principle). Outcome data obtained from all participants are included in the data analysis, regardless of protocol adherence. Per protocol analysis will be performed as sensitivity analysis. If there is missing data on the primary outcome, a multiple imputation method will be used.

#### Plans to give access to the full protocol, participant level-data and statistical code {31c}

Only the research members will have access to the trial files. After the completion of the study, the results will be made public through publication in a scientific journal along with conferences related to ear, nose and throat, as well as the ClinicalTrials.gov website. The data generated or analysed during this study will be available from the corresponding author on reasonable request. The protocol will be sent to a journal for publication.

### Oversight and monitoring

#### Composition of the coordinating centre and trial steering committee {5d}

The research group is responsible for participant safety, study design, database integrity and study conduct. The group as a whole and particularly its leader (O.-P.A.) and statisticians (E.L., P.O.) have long experience in observational and interventional clinical trials. The group has wide clinical experience on the medical condition being studied. The group deals with any clinical or scientific problems together. The group leader is primarily responsible for the ethical aspects of this project including data management and storage. Oulu University Hospital’s administrative leader has granted permission to perform this research in the hospital.

#### Composition of the data monitoring committee, its role and reporting structure {21a}

This trial includes only conventional treatments so trial participation specifically exposes subjects to no extra risk of any complication. Therefore, this project has no data monitoring committee.

#### Adverse event reporting and harms {22}

Trial participants are informed about the risks involved in the surgical procedures. These mainly include postoperative bleeding, postoperative infections and pain. The study participants will be recruited from a group of patients that would usually be operated on at our clinics according to common practice without any research setting, so the study itself does not add any risk for the participants as the novel TT seems to have fewer complications than the traditional TE [23]. In case of complications, participants are instructed to contact their respective clinic and their care is arranged by the study hospitals according to good clinical practice. All study personnel are employees of the trial hospital and will be insured by their employer.

We collect data about potential harms and will report our findings. We collect data from the patients about the adverse effects. Severe harms are also recorded from patient files.

#### Frequency and plans for auditing trial conduct {23}

Auditing is not planned.

#### Plans for communicating important protocol amendments to relevant parties {25}

All research group members may introduce protocol amendments. These are then considered together, and the principal investigator will be responsible for the final decision to amend and how the substantive changes are communicated to the relevant stakeholders (the Northern Ostrobothnia Hospital District’s Ethics Committee and ClinicalTrials.gov register). The protocol version with a date and list of amendments is clearly presented in the protocol.

#### Dissemination plans {31a}

After the completion of the study, the results will be made public through publication in a scientific journal, at conferences related to ear, nose and throat diseases and on the ClinicalTrials.gov website.

## Discussion

This protocol deals with a multicentre, partly blinded, randomised, parallel-group clinical trial that explores the QoL change 6 months after tonsillectomy (TE) versus tonsillotomy (TT) versus watchful waiting (WW) among adult patients suffering from recurrent or chronic tonsillitis. Theoretically, the structure of the palatal tonsils changes in infective tonsillar diseases, and removal of all or part of this altered lymphoid tissue alleviates the patients’ symptoms and improves QoL. Special reasons for the tonsillar surgery like malignant tumours, the presence of cardiac valvular disease associated with recurrent streptococcal infections or recurrent febrile seizures are relatively rare and most of the tonsillar resections are done on relatively young adults on order to improve their QoL.

Randomised trials have presented information to the effect that, in adults with recurrent tonsillitis, TE results in fewer symptoms and further episodes [24, 25]. The most common harm related to TE is postoperative throat pain. Still, the net change in days with throat pain decreased in the TE group as compared to the WW group. TT has been shown to result in similar QoL improvement as TE in adults with infective or obstructive tonsillar disease [7, 8]. However, these trials have lacked a control group. To our knowledge, the effects of TE and TT on QoL in adults with recurrent or chronic tonsillitis have not been investigated in a randomised controlled trial. Because of this, the role of TE and TT in the treatment of these diseases has been somewhat vague in international guidelines [4, 26]. The trial we have started will give more accurate estimates of whether tonsil surgery improves the QoL in adults with recurrent of chronic tonsillitis and whether the lighter TT is as effective as TE.

There are some strengths and limitations regarding our study. As practically, all tonsillar surgery in the area is done in the contributing hospitals, the participant sample is population-based. Prior to this study, we also conducted a matched cohort study in the same area with similar entry criteria. There, we translated and validated the German TOI-14 instrument in Finnish and explored the interpretation of different scores according to the recommendations of the (ISOQOL) and COSMIN initiative [15–17]. We found that the Finnish instrument showed good reliability, content and construct validity, as well as responsiveness. The results of this study also provide a basis for the sample size calculation and statistical analysis for this trial. To increase the generalisability of the results, we have a multicentre trial where a variety of surgeons from residents to experienced specialists perform the surgical procedures under study. The fact that this is partly (TE+TT vs. WW) an open-label trial constitutes a limitation. Knowledge of the intervention may cause detection bias in the measurement of outcomes and exclusion/attrition bias in the decision to withdraw from the trial. The wait time for tonsillectomy is restricted by Finnish law to no more than 6 months, which resulted in a relatively short follow-up. However, we think that the short-term effect of tonsillectomy shows its overall usefulness. Earlier research in children has shown that the objective outcomes after tonsillectomy do not depend on the length of follow-up [27].

### Trial status

Recruitment began on December 9, 2020, and is currently ongoing. We anticipate it to end in 2023, although it slowed somewhat in the spring of 2021 because of the coronavirus pandemic.

## Supplementary Information

**Additional file 1.** ‘List of Variables’ and contains the list of all variables we have in this study, timing of the variable and who records the variable.

**Additional file 2.** ‘Funding documentation’ and contains information of funding of this study.

**Additional file 3.** Is a translated approval statement of the study from Northern Ostrobothnian Hospital District’s Ethical Committee.

## Data Availability

The datasets used and/or analysed during the current study are available from the corresponding author on reasonable request.

## Abbreviations

QoL: Quality of life
RAND-36: Research and Development 36-item Health Survey
SD: Standard deviation
TOI-14: Tonsillectomy Outcome Inventory-14
TE: Tonsillectomy
TT: Tonsillotomy
WW: Watchful waiting
ISOQOL: International Society for Quality-of-Life Research
COSMIN: Consensus-based Standards for the Selection of Health Measurement Instruments
CONSORT: Consolidated Standards of Reporting Trials
SPIRIT: Standard Protocol Items: Recommendations for Interventional Trials
