# Correction: Total or partial tonsillar resection (tonsillectomy or tonsillotomy) to change the quality of life for adults with recurrent or chronic tonsillitis: study protocol for a randomised controlled trial

**DOI:** 10.1186/s13063-023-07627-z

**Published:** 2023-10-29

**Authors:** Aleksi Laajala, Paulus Tokola, Timo J. Autio, Timo Koskenkorva, Mikko Tastula, Pasi Ohtonen, Esa Läärä, Olli-Pekka Alho

**Affiliations:** 1 Department of Otorhinolaryngology and Head and Neck Surgery, Oulu University Hospital, P.O. Box 5000, N90014 Oulu, Finland; 2 PEDEGO Research Unit, University of Oulu, Oulu, Finland; 3 Medical Research Center Oulu, Oulu, Finland; 4 Division of Operative Care, Oulu University Hospital, Oulu, Finland; 5 Research Unit of Mathematical Sciences, University of Oulu, Oulu, Finland

**Correction: BMC Trials 22, 617 (2021)**

**https://doi.org/10.1186/s13063-021-05539-4**

Following publication of the original article [1], we have been informed of an error calculation in the “Sample size {14}” paragraph in “Methods: Participants, interventions and outcomes” section.

The authors would like to emphasize that they have not altered the key parameters of the sample size calculation that must be determined and registered before the trial begins. The parameters of alfa error = 0.05, beta error = 0.1, standard deviation = standard deviation and noninferiority limit = 10 on the Tonsillectomy Outcome Inventory Scale (TOI-14), that the authors found to be the minimum change a patient could sense, have all been determined at the planning phase of the trial. In this correction w these parameters are not changed, rather the calculation error made when the data on the linear scale was log − transformed because of the nonnormality is addressed. The previously published wrong sample size calculations were based on a wrong standard deviation and on a wrong noninferiority limit on the log-scale resulting in a substantial error in the number of patients needed in each group.

- Originally published sample size paragraph:

“Our principal outcome is a disease-specific QoL questionnaire TOI-14 score at 6 months of follow-up. According to Laajala et al. [17], a difference of 10 points is clinically significant. Further, the TOI-14 score was detected to be highly skewed to the right with excess zeroes at 6 months of follow-up, so we used a logtransformation (log (1 + TOI-14)) in sample size calculations. Our hypotheses were (1) both surgically treated groups (TE + TT) were superior (1.50 vs 2.71, SD = 1.0) compared to the follow-up (WW), and (2) TT is noninferior to TE (1.50 vs 1.50, SD = 1.0 with non-inferiority marginal = 0.41). In both calculation α = 0.05 and β = 0.10 (power = 0.90). According to this, (1) 15 patients and (2) 102 patients per group will be needed. To ensure that we have adequate power for the follow-up group, we decided to recruit 51 patients into that group. Further assuming a drop-out rate of 10%, the sample size for surgically treated groups is 114 and for the follow-up group 57 patients (altogether 285). Sample size estimation was performed only for the principal outcome, and other comparisons are hypothesis generating only.”

- Corrected sample size paragraph:

“Our principal outcome is a disease-specific QoL questionnaire TOI-14 score measured at baseline and at 6 months of follow-up. According to Laajala et al. [17], a difference of 10 points is clinically significant. Further, the TOI-14 score was detected to be highly skewed to the right with excess zeroes at 6 months of follow-up, so we used a natural logarithmic transformation (log (1+TOI-14)) in sample size calculations. Our hypotheses were (A) both surgically treated groups (TE+TT combined) are superior (mean 1.6 vs 3.0, SD=1.0) compared to the follow-up (WW), and (B) TT is noninferior to TE (change score mean 3.1, SD=0.7 with non-inferiority limit=0.4). In both calculations α=0.05 and β=0.10 (power=0.90). According to this and taking into consideration the allocation ratio, (A) 7 and 28 patients in the WW and the combined TE+TT groups, respectively, and (B) 53 patients in the TE and the TT groups will be needed. Considering the allocation ratio WW:TE:TT = 1:2:2 and ensuring adequate sample size for each group, we decided to recruit 27 patients into the WW group and 53 in both the TE and the TT groups. Further assuming a drop-out rate of 10%, the sample size for both surgically treated groups is 59 and for the follow-up group 30 patients (altogether 148). Sample size estimation was performed only for the principal outcome, and other comparisons are hypothesis generating only.”

The original article has been corrected.
