# Supplementary material for: Total or partial tonsillar resection (tonsillectomy or tonsillotomy) to change the quality of life for adults with recurrent or chronic tonsillitis: study protocol for a randomised controlled trial
Source: Trials. 2021 Sep 15;22:617. doi: 10.1186/s13063-021-05539-4 (PMC8441038; doi:10.1186/s13063-021-05539-4)
Supplement: Supplementary file 2 — Additional file 2. ‘Funding documentation’ and contains information of funding of this study. [file 13063_2021_5539_MOESM2_ESM.pdf]

FUNDING DOCUMENTATION

This is an investigator-initiated study. The participants' communities finance the clinical visits and treatments with a small deductible paid by the participants. The hospital and university pay the participating physicians' salaries as usual and they do the research work as part of their daily work. The more extensive scientific work done mainly by the doctoral student Aleksi Laajala is covered with governmental financing granted for this research project for 2021 and sought for the years 2022-2023. Otherwise, we receive no financial, material, or other support for this work.

Below, please find the funding decision of the Oulu University Hospital District's Research Steering Committee (in Finnish). On page 3, it is shown that 20 000 euros are granted from the governmental financing for professor Olli-Pekka Alho's research project K 53760 for the year 2021.

VALTION TUTKIMUSRAHOITUS OYS-ERVALLE VUOSILLE 2021 - 2022							
**Rahoitus-ryhmä** (1-4)	**Organi-saatio**	**Tutkimushanke** (koko nimi)	**Hakija**	**Haettu rahoitus 2021**	**Haettu rahoitus 2022**	Valtion tutkimus-rahoitus 2021	Valtion tutkimus-rahoitus 2022
1	PPSHP/ Lapset ja nuoret	Vastasyntyneen aivojen lääkkeellinen suojaaminen synnytyksessä esiintyneen hapenpuutteen hoidossa	Haapsamo Mervi	14 709	17 570	10 000	
1	PPSHP/ Lapset ja nuoret	Kuumekouristusten patomekanismit	Helander Heli	20 000	20 000	10 000	
1	PPSHP/ Lapset ja nuoret	Perintö- ja ympäristötekijöiden vaikutukset ennenaikaisesti syntyneiden lasten neurologiseen ja neurokognitiiviseen	Kaukola Tuula	58 242	68 559	10 000	
1	PPSHP/ Lapset ja nuoret	Lasten ruoka-allergian hoitomenetelmät ja primaaripreventio	Kulmala Petri	40 000	40 000	20 000	
4	PPSHP/ Lapset ja nuoret	Hedelmöittymisen sekä alkio- ja sikiökehityksen molekyylimekanismit	Lehtonen Siri	15 000	25 000	10 000	
1	PPSHP/ Lapset ja nuoret	Geneettiset sairaudet Pohjois-Suomessa	Moilanen Jukka	26 500	27 000	10 000	
1	PPSHP/ Lapset ja nuoret	Lihavuus ja lisääntyminen	Morin-Papunen Laur	80 000	80 000	20 000	
1	PPSHP/ Lapset ja nuoret	PEPPI-tutkimus: Istukan toimintahäiriölle alttiiden äitien identifioiminen ensimmäisen ja kolmannen raskauskolmanneksen aikana	Nevalainen Jaana	65 200	76 300	10 000	
1	PPSHP/ Lapset ja nuoret	Menopaussi-iän ja hormonaalisten tekijöiden terveysvaikutukset naisilla	Niinimäki Maarit	40 000		10 000	
1	PPSHP/ Lapset ja nuoret	Lapsuusiän syöpien kehittyminen, hoitoon liittyvät haittavaikutukset ja syöpähoidon kehittäminen	Niinimäki Riitta	40 000	40 000	20 000	
4	PPSHP/ Lapset ja nuoret	D-vitamiinin terveysvaikutukset keskosena syntyneessä väestössä	Ojaniemi Marja	55 000	47 000	20 000	
3	PPSHP/ Lapset ja nuoret	Deksemedetomidiinin teho ja vaikutukset lasten toimenpiteiden aikaisena kipua hoitavana ja rauhoittavana lääkityksenä	Peltoniemi-Ailisto Outi	15 400	17 200	10 000	
1	PPSHP/ Lapset ja nuoret	Common symptoms translate into severe health risks in women -promoting diagnostics, treatment modalities and awareness of PCOS	Piltonen Terhi	80 000	80 000	60 000	
3	PPSHP/ Lapset ja nuoret	Näyttöön perustuvat yhtenäiset käytännöt: teknologiasta vaikuttavuutta potilaan hoitoon	Pölkki Tarja	65 200	60 100	20 000	
3	PPSHP/ Lapset ja nuoret	Aistivammaisuuden geneettiset taustatekijät Pohjois-Suomessa	Rahikkala Elisa	10 000		10 000	
1	PPSHP/ Lapset ja nuoret	Yleisimpien perinataalikauden sairauksien mekanismit, enneltaehkäisy ja hoito	Rämet Mika	80 000	80 000	30 000	
3	PPSHP/ Lapset ja nuoret	Lasten tapaturmien, sairauksien ja kehityshäiriöiden riskitekijät ja tulokset sekä uudet kirurgiset hoitomuodot	Sinikumpu Juha-Jaak	43 500	43 500	20 000	
1	PPSHP/ Lapset ja nuoret	Raskauden ja synnytyksen aikaisen antibioottialtistuksen ja lapsen mikrobiomin merkitys lapsen myöhemmälle terveydelle: Rekisteritutkimus ja koneoppimismalli	Tapiainen Terhi	75 000	48 500	40 000	
1	PPSHP/ Lapset ja nuoret	Liikunta ja ravitsemus Tyypin 1 diabetesta sairastavilla lapsilla ja nuorilla	Tossavainen Päivi	58 341	54 841	20 000	

1	PPSHP/ Lapset ja nuoret	Lapsuusiän kehityshäiriöiden, epilepsioiden ja liikehäiriöiden uudet diagnostiikkamenetelmät ja hoitomuodot	Uusimaa Johanna	80 000	80 000	20 000	
1	PPSHP/ Lapset ja nuoret	DIPP - Tyypin 1 diabeteksen ennustaminen ja ehkäisy	Veijola Riitta	80 000	80 000	80 000	80 000
1	PPSHP/ Lapset ja nuoret	Lastenreuman taudinaktiivisuuden arviointi ja sen ennustetyökalut	Vähäsalo Paula	30 000	30 000	20 000	
1	PPSHP/ Lapset ja nuoret	Finngedi-tutkimus - raskausdiabeteksen seulonta, menetelmät ja merkitys äidin ja lapsen pitkäaikaisterveydelle	Vääräsmäki Marja	80 000	80 000	60 000	
3	PPSHP/ Medisiininen	Monisairastavuuden profiilit väestössä, tunnistaminen perusterveydenhuollossa ja vaikutukset elämänlaatuun ja terveydenhuollon kustannuksiin	Auvinen Juha	80 000	80 000	70 000	70 000
3	PPSHP/ Medisiininen	Endokriinisten sairauksien tutkimusryhmä	Ebeling Tapani	23 000	23 000	20 000	
4	PPSHP/ Medisiininen	Infektioalttiuden ja immuunipuutossairauksien genetiikka ja biologia	Hautala Timo	76 784	76 784	30 000	30 000
4	PPSHP/ Medisiininen	Atooppisen ekseeman riskitekijät	Huilaja Laura	80 000	80 000	44 000	40 000
1	PPSHP/ Medisiininen	Metabolisen syndrooman syntyyn, ehkäisyyn ja hoidon onnistumiseen vaikuttavat tekijät	Hukkanen Janne	80 000	80 000	30 000	
1	PPSHP/ Medisiininen	Sydänperäisen äkkikuoleman erityispiirteet ja genetiikka	Junttila Juhani	80 000	80 000	80 000	80 000
2	PPSHP/ Medisiininen	Interstitiaalisten keuhkosairauksien tutkimus	Kaarteenaho Riitta	60 000	60 000	40 000	
1	PPSHP/ Medisiininen	Väestöpohjainen tutkimus, jossa selvitetään Suomalaisten levinneeseen suolistosyöpään sairastuneiden taudin kliinistä käyttäytymistä, etäpesäkirurgian mahdollisuutta ja elinaikaa sekä prognostisia ja prediktiivisiä biomarkkereita (RAXO)	Kallio Raija	45 000	45 000	10 000	
1	PPSHP/ Medisiininen	Metformiinivälitteinen oksidatiivinen stressi kiinteiden kasvainten karsinogeneesissä ja ennusteessa. Epidemiologinen ja translationaalinen tutkimus	Karihtala Peeter	40 000	41 000	20 000	
3	PPSHP/ Medisiininen	COVID-19-tauti ja siihen assosioituvat tekijät Millenium-kohortin reumasairauspotilailla taustaväestöön verrattuna	Karjalainen Anna	10 000	10 000	10 000	
1	PPSHP/ Medisiininen	Syövän varhaisdiagnostiikan ja hoidon kehittäminen, erityiskohteena keuhkosyöpä, immuno-onkologiset lääkehoidot ja elektroniset lähestymistavat	Koivunen Jussi	70 000	65 000	10 000	
3	PPSHP/ Medisiininen	Syövän diagnostiikan toteutuminen ja hoidon vaikuttavuus, sekä potilaan ja lääkärin näkemykset hoidon tehosta	Kuittinen Outi	30 000	30 000	20 000	
4	PPSHP/ Medisiininen	Silmänpohjalöydökset kardiovaskulaarisairauksissa	Lehtola Heidi	20 000	20 000	10 000	
1	PPSHP/ Medisiininen	Mitokondrioiden osuus Parkinsonin taudin synnyssä	Majamaa Kari	80 000	80 000	50 000	
1	PPSHP/ Medisiininen	Interventiokardiologian tutkimus: toimenpiteiden optimointi, toimenpiteiden komplikaatiot, parhaan hoidon valinta, seurannat ja etiologinen tausta	Mäkikallio Timo	80 000		20 000	

1	PPSHP/ Medisiininen	Glymfaattiset aivopulsaatiot ja sädehoidon vaikutukset glioblastoma multiformea sairastavissa potilaissa	Nikkinen Juha	40 000	40 000	20 000	
1	PPSHP/ Medisiininen	Sydänsähkökäyrän repolarisaation ajallisen vaihtelun ja P-aallon poikkeavuuksien ennusteellinen merkitys	Perkiömäki Juha	80 000	80 000	30 000	
1	PPSHP/ Medisiininen	Työikäisten kognitiiviset heikentymät ja muistisairaudet	Remes Anne	80 000	80 000	70 000	70 000
1	PPSHP/ Medisiininen	Autoimmunisaation riskitekijät ja mekanismit pemfigoidissa	Tasanen-Määttä Kais	80 000	80 000	70 000	70 000
1	PPSHP/ Medisiininen	Sydän- ja verisuonisairauksiin, muistihäiriöihin ja aineenvaihduntapoik-keavuuksiin vaikuttavat tekijät keski-iästä vanhuuteen	Ukkola Olavi	80 000	80 000	20 000	
1	PPSHP/ Operatiivinen	Tehohoitotutkimus	Ala-Kokko Tero	80 000	80 000	50 000	
3	PPSHP/ Operatiivinen	Endoskooppinen sivuontelokirurgia toistuvissa akuuteissa nenä-ja sivuontelotulehduksissa - satunnaistettu kontrolloitu tutkimus	Alakärppä Antti	8 000	8 000	8 000	
3	PPSHP/ Operatiivinen	Nielurisojen koko- ja osapoiston vaikutus toistuvia tai kroonista nielurisatulehdusta sairastavien aikuisten elämänlaatuun: satunnaistettu, kontrolloitu, kliininen monikeskustutkimus	Alho Olli-Pekka	22 600	22 600	20 000	
1	PPSHP/ Operatiivinen	Uudet menetelmät diabeettisen retinopatian seulonnassa ja hoidossa	Hautala Nina	40 000	35 000	20 000	
1	PPSHP/ Operatiivinen	Mahasuolikanavan syövät ja immuunivaste	Helminen Olli	55 000	55 000	20 000	
1	PPSHP/ Operatiivinen	Ruokatorvi- ja haimasyövän diagnostiikan tehostaminen ja uusien biomarkkereiden löytäminen	Huhta Heikki	80 000	80 000	10 000	
3	PPSHP/ Operatiivinen	Alaselkäkivun biopsykososiaalisen hoitomallin kustannusvaikuttavuus perus-terveydenhuollossa ja työterveyshuollossa. Monisairastavuuden merkitys selkäkivusta johtuvaan työkyvyttömyyteen	Karppinen Jaro	79 985		70 000	70 000
1	PPSHP/ Operatiivinen	Hapenpuute ja sen vaikutukset sydänkirurgiassa kokeellisella tutkimusmallilla	Kiviluoma Kai	80 000	80 000	20 000	
1	PPSHP/ Operatiivinen	Lihavuusleikkauksen vaikutus toimintakykyyn, nivelrikon radiologiseen etenemiseen sekä geenien ilmentymisen säätelyyn. Mukoosan ja	Koivukangas Vesa	20 000	20 000	10 000	
1	PPSHP/ Operatiivinen	Elämänlaatu ja ennuste pään ja kaulan syövässä	Koivunen Petri	67 680	67 680	20 000	
4	PPSHP/ Operatiivinen	Vatsakalvolle levinneen paksu- ja peräsuolisuolisyövän tutkimusryhmä: Sytoreduktiivinen kirurgia, HIPEK-hoito ja peritoneealikarsinoosin syntymekanismit	Koskela Marjo	19 000	19 000	10 000	
3	PPSHP/ Operatiivinen	Hampaiden ja suun terveys osana yksilön, erityisryhmien ja koko väestön terveyttä ja hyvinvointia	Laitala Marja-Liisa	80 000	80 000	30 000	
1	PPSHP/ Operatiivinen	Tupakkatuotteiden ja nikotiinin soluvaikutukset paranemisessa ja nivelreumassa	Lehenkari Petri	45 000	45 000	40 000	
1	PPSHP/ Operatiivinen	Ortopedian yhteiset	Leppilahti Juhana	45 000		10 000	

1	PPSHP/ Operatiivinen	Pohjois-Suomen syntymäkohortin silmätutkimukset 2	Liinamaa Johanna	40 000	40 000	10 000	
1	PPSHP/ Operatiivinen	Toipuminen ja elämänlaatu operatiivisen hoidon jälkeen	Liisanantti Janne	80 000	80 000	20 000	
3	PPSHP/ Operatiivinen	Hampaiden kehitys, puhkeaminen ja geneettinen tausta	Lähdesmäki Raija	55 500	24 500	10 000	
4	PPSHP/ Operatiivinen	Lantionpohjan toimintahäiriöiden diagnostiikka, lantion takaosan laskeumien leikkaustekniikat ja robottikirurgian vaikuttavuus	Mäkelä-Kaikkonen Jo	10 000	10 000	10 000	
1	PPSHP/ Operatiivinen	Tekonivelkirurgian tutkimusryhmä	Niinimäki Tuukka	20 000	20 000	10 000	
1	PPSHP/ Operatiivinen	Purentavirheiden moderni diagnostiikka, geneettinen tausta ja yhteydet terveystekijöihin Pohjois-Suomalaisissa kohorttiaineistoissa	Pirttiniemi Pertti	80 000		30 000	
3	PPSHP/ Operatiivinen	Modernin verisuonikirurgian tutkimusyksikkö	Pokela Matti	10 000	10 000	10 000	
1	PPSHP/ Operatiivinen	Prevention of incisional and parastomal hernias; PRELOOP, CHIMNEY and PREEMER trials	Rautio Tero	27 000	35 000	27 000	
4	PPSHP/ Operatiivinen	Milloin päivystyksellinen gastrokirurginen erikoissairaanhoito on kuolinprosessiin puuttumista?	Rintala Jukka	20 000	20 000	10 000	
1	PPSHP/ Operatiivinen	Glaukoomamuutokset pohjoissuomalaisissa syntymäkohorteissa - glaukooman ja silmä-sairauksien seulonnan kustannusvaikuttavuus	Saarela Ville	80 000	80 000	20 000	
1	PPSHP/ Operatiivinen	Vajaaravitsemusta korjaavien toimenpiteiden, sarkopenia ja suolen mikrobiomin merkitys paksusuolen syövässä (NutriBac -studies)	Saarnio Juha	80 000	80 000	20 000	
1	PPSHP/ Operatiivinen	Kielisyövän uusien hoitomuotojen etsiminen ja menetelmien kehittäminen henkilökohtaisen hoitovasteen arviointiin	Salo Tuula	80 000	80 000	80 000	80 000
1	PPSHP/ Operatiivinen	Purentaelimistön kipujen ja toimintahäiriöiden taustatekijät ja uuden kansainvälisen diagnoosikriteeristön soveltuvuus suomalaisilla potilailla	Sipilä Kirsi	76 000	76 000	10 000	
4	PPSHP/ Operatiivinen	Tupakkatuotteiden ja nikotiinin haittavai-kutukset - näkökulmana sekä väestötutkimus, kliininen tutkimus että in vitro tutkimus	Tanner Tarja	40 000	40 000	10 000	
1	PPSHP/ Operatiivinen	Yleisimpien urologisten sairauksien epidemiologia, diagnostiikka ja hoito	Vaarala Markku	50 000	50 000	10 000	
3	PPSHP/ Operatiivinen	Parodontaalisairaudet ja yleisterveys	Ylöstalo Pekka	50 000	50 000	30 000	
3	PPSHP/ Psykiatria	Aivot, mieli ja kehitys	Ebeling Hanna	48 000	31 000	30 000	
3	PPSHP/ Psykiatria	Mielenterveyskuntoutujien asumispalveluiden laatu ja kustannukset: tiedonlähteenä kansalliset tilastotietokannat ja empiirinen aineisto asumispalveluyksikköjen	Hakko Helinä	20 000	22 000	10 000	
1	PPSHP/ Psykiatria	Psykoosien elinaikainen kulku ja psykoosilääkkeiden vaikutukset	Jääskeläinen Erika	80 000	80 000	50 000	

3	PPSHP/ Psykiatria	Mielenterveyshäiriöiden yhteys nuorten aikuisten elämänlaatuun, terveyteen, liikennekuolemiin ja itsetuhoisuuteen : Alaikäisten nuorten potilaskohortti-, sekä epidemiologinen normaaliväestön	Riipinen Pirkko	45 000	45 000	20 000	
1	PPSHP/ Psykiatria	Psyykkisesti tai somaattisesti sairas vanhempi ja lapsen mielenterveys - riskiryhmien tunnistaminen, prevention kohderyhmät ja menetelmät	Räsänen Sami	36 800	36 800	10 000	
1	PPSHP/ Psykiatria	Maternal Immune Activation (MIA) and Mental health of the offspring in the Northern Finland Birth Cohort 1986 (NFBC 1986)	Veijola Juha	80 000	80 000	70 000	70 000
1	PPSHP/ Sairaanhoid.palvelut	Glymfaattinen diagnostiikka ja ohjattu terapia keskushermoston sairauksissa	Kiviniemi Vesa	80 000	80 000	40 000	
4	PPSHP/ Sairaanhoid.palvelut	Veden ja hemodynamiikan mittaus aivokudoksesta aivosyövän hoidossa sädehoidon aikana	Korhonen Vesa	37 000	37 000	20 000	
1	PPSHP/ Sairaanhoid.palvelut	GICAN2020: Mahasuolikanavan kasvaimet, niihin liittyvät tilat ja diagnostisten menetelmien kehitys	Mäkinen Markus	80 000	80 000	60 000	
4	PPSHP/ Sairaanhoid.palvelut	Polvinivelrikon nykyaikainen kuvantaminen	Nevalainen Mika	50 000	50 000	20 000	
1	PPSHP/ Sairaanhoid.palvelut	Uudet magneettikuvausmenetelmät nivelruston tutkimiseksi	Nieminen Miika	80 000	80 000	60 000	
1	PPSHP/ Sairaanhoid.palvelut	Magneettikuvantaminen alaselkäkivun ja rintasyövän diagnostiikassa ja hoidon seurannassa, tekoälyn käyttö magneetti-kuvantamisen jälkikäsittelyssä ja analyysissä sekä kuvantamisen turvallisuuskulttuurin tutkimus	Niinimäki Jaakko	79 969		40 000	
1	PPSHP/ Sairaanhoid.palvelut	Targeted treatment for the cartilage endplate (CEP) in intervertebral disc (IVD) degeneration by understanding of the phenotype of CEP chondrocytes	Tervonen Osmo	80 000	80 000	10 000	
4	PPSHP/ Sairaanhoid.palvelut	Seuraavan sukupolven turvallisia ja tietoteknisiä keinotekoisia älykkyys menetelmiä lääketieteelliseen kuvantamiseen	Tiulpin Aleksei	79 841	77 741	20 000	
3	Hoitotiede	Digiohjauksen edellytykset, prosessi ja vaikutukset hoitopoluilla: potilaiden, henkilöstön ja kustannusten näkökulmat	Kyngäs Helvi	80 000	80 000	40 000	
2	Konserni-palvelut	Tiedon hyödyntäminen potilashoidon ja palvelujärjestelmän hyväksi	Meriläinen Merja	28 700	25 000	10 000	
2	Konserni-palvelut	Lääkehoidon tehon ja turvallisuuden parantaminen	Turpeinen Miia	80 000	80 000	40 000	
1	PTH-yksikkö	Biopsykososiaalisten tekijöiden vaikutus sokeriaineenvaihduntaan, mielenterveyteen ja aivoterveyteen elämänkulun aikana	Timonen Markku	80 000	80 000	40 000	
4	Biopankki	Seerumin D-vitamiinitasot raskaana olevilla naisilla Suomessa: Vuodenaikavaihtelu ja D-vitamiinilla täydennettyjen elintarvikkeiden vaikutus 1983 - 2016	Öhman Hanna	49 000	30 000	10 000	
3	LPSHP	Rekisteriseuranta avoimen dialogin periaattein toteutetun nuorisopsykiatrisen hoidon hoitotuloksista	Bergström Tomi	20 000	20 000	10 000	
1	Nordlab	Hoitokäytännöt ja laboratoriotutkimukset hematologiassa	Savolainen Eeva-Riit	80 000	80 000	10 000	
1	Nordlab	Perinnöllinen alttius rintasyöpään: uudet tekijät, solumekanismit, tautimallintaminen ja kliininen merkitys	Winqvist Robert	80 000	80 000	60 000	

3	Nordlab	Laboratoriotutkimusten käytön optimointi ja laboratoriotulosten toisiohyödyntäminen terveyspalvelujen kehittämisessä ja arviossa	Männistö Tuija	25 000		10 000	
3	Oulun kaupunki	Geriatrinen hammaslääketiede	Syrjälä Anna-Maija	50 000	50 000	10 000	
3	Rovaniemen kaupunki	Perusterveydenhuollon tyypin 2 diabetes-potilaiden hoitotasapainoon vaikuttavat tekijät	Hagnäs Maria	20 000	20 000	10 000	
1	Tornion kaupunki	Suoli-aivoakseli ja mahasuolikanavan sairaudet väestössä	Ronkainen Jukka	80 000	80 000	20 000	
OYS-erva yhteensä						2 599 000	660 000
VALTION TUTKIMUSRAHOITUS OYS-ERVALLE V. 2021 - 2022 YHTEENSÄ							
OYS-erityisvastuualueen organisaatiot				Vuosi 2021	Vuosi 2022		
Pohjois-Pohjanmaan sairaanhoitopiirin ky				2 469 000	660 000		
Länsi-Pohjan sosiaali- ja terveyspalvelujen ja sairaanhoitopiirin ky				10 000			
Oulun kaupunki				10 000			
Rovaniemen kaupunki				10 000			
Tornion kaupunki				20 000			
Pohjois-Suomen laboratoriokeskuksen liikelaitoskuntayhtymä Nordlab				80 000			
OYS-erva yhteensä				2 599 000	660 000		
