# Supplementary material for: Total or partial tonsillar resection (tonsillectomy or tonsillotomy) to change the quality of life for adults with recurrent or chronic tonsillitis: study protocol for a randomised controlled trial
Source: Trials. 2021 Sep 15;22:617. doi: 10.1186/s13063-021-05539-4 (PMC8441038; doi:10.1186/s13063-021-05539-4)
Supplement: Supplementary file 3 — Additional file 3. Is a translated approval statement of the study from Northern Ostrobothnian Hospital District’s Ethical Committee. [file 13063_2021_5539_MOESM3_ESM.docx]

Ethical Approval Document translated from original copy of 31st July 2020

Regional ethical committee of Northern Ostrobothnia Hospital District Meeting date 18^th^ May 2020

Number of application: EETTMK: 46/2020

113§ Total or partial tonsillar resection to change quality of life for adults with recurrent or chronic tonsillitis

**Application for Approval**

Clinical treatment/ intervention study, which will be conducted as controlled, randomised and blinded. Supervisor involved in study is professor Olli-Pekka Alho, Oulu University hospital (OYS)

Research centres and researchers

Laajala A, MD, doctoral student, specialist in Otorhinolaryngology, OYS
Tokola P, MD, doctoral student, specialist in Otorhinolaryngology, OYS
Autio T, MD, PhD, specialist in Otorhinolaryngology, OYS
Koskenkorva T, MD, PhD, specialist in Otorhinolaryngology, OYS Tastula M, MD, specialist in Otorhinolaryngology, OYS Ohtonen P, MSc, statistician, OYS
Läärä E, professor of Statistics, University of Oulu Alho O-P, MD, PhD, professor of Otorhinolaryngology, OYS Kuoppala P. MD, specialist in Otorhinolaryngology, Seinäjoki Central Hospital Mäkinen J, MD,PhD.specialist in Otorhinolaryngology, Lapland Central Hospital Weitz-Tuoretmaa A. MD. specialist in Otorhinolaryngology, Keski-Pohjanmaa Central Hospita Kristo L. MD. specialist in Otorhinolaryngology, Länsi-Pohja Central Hospital

Researcher driven study

Documents provided to the committee: Application for approval (4.5.2020), Summary of study design (version 2, 29.4.2020), Study design (version 6/13.4.2020), Statement of ethical aspects from supervisor involved in study(4.5.2020), Patient information form(version1/14.4.2020), Consent form(version3/15.4.2020) Description of data protection in scientific study and estimation of effect on data protection(4.5.2020), Background information form in CureLisa(4.5.2020), Demographic and background information form in Terveyskylä (Version3/29.4.2020), Patient information form after surgery(4.5.2020), TOI-14 questionnaire (4.5.2020), RAND36 questionnaire(4.5.2020), Symptom diary(version6/29.4.2020), Pre- and postoperative anchorquestions(4.5.2020), postoperative information form after six months(4.5.2020)

**Proposition for decision**

Ethical committee decides

1. on a matter in hearing
2. to collect statement fee of 0€ (Social and Health ministry statute 1287/2018, 1 § 2 mom)

18.5.2020 Eettmk § 113 Application was presented by Professor Jyrki Mäkelä.

Ethical committee asks to do following changes and corrections:

Patient information form:

-The first paragraph of the sections of Purpose of the study should be revised and clarify, for example: “The purpose of the study is… adult patients’ quality of life compared to quality of life of patient undergoing surgery after six months follow-up.” In addition, it is asked to say what is conventional surgery (total or partial tonsillectomy). In addition, it is asked to correct a misspelling.

Consent form should be revised as follows: “..information can be searched from patient ~~database~~ information systems and can be used in this study.”

Ethical committee asks to clarify, how patient data is moved between hospitals. In addition, committee informs, that after 1^st^ of April, Findata is the only competent official to permit combining of different registries.

Ethical committee recommends replacing the word follow-up group with a term delayed operation group.

Otherwise, if completed as planned, this study fulfils the conditions of the law about medical research (488/1999).

**Decision**

Ethical committee decides

1. to approve the study design, given that corrections and clarifications pointed out by committee are done.

Committee authorized the chairman and secretary of the committee to check and approve these corrections.

This study can’t be started before corrections pointed out by the committee are done and approved

1. to collect statement fee as proposed.

Pasi Ohtonen, member of the committee and study group, left the meeting during processing and decision making for this application

29.7.2020 Corrections and clarifications pointed out by the committee are done. Background forms and symptom diary have also been edited.

Documents provided to the committee: e-mail (Laajala/16^th^ and 26^th^ of June 2020), Summary of study design (version 3, 16.6.2020), Study design (version 7/16.6.2020), Patient information form(version2/8.6.2020), Consent form(version4/16.6.2020), Background information form in CureLisa(16.6.2020), Demographic and background information form in Terveyskylä (Version4/16.6.2020), Symptom diary application(version8/16.6.2020)

**Decision:** Approved and labelled for notification

Jyrki Mäkelä Minna Mäkiniemi

Head of ethical committee Secretary of ethical committee
